# Analysis of the Effect of the Surface Inclination Angle on the Roughness of Polymeric Parts Obtained with Fused Filament Fabrication Technology

**DOI:** 10.3390/polym15030585

**Published:** 2023-01-23

**Authors:** Francisco Martín Fernández, María Jesús Martín Sánchez

**Affiliations:** Department of Civil, Materials, and Manufacturing Engineering, School of Industrial Engineering, University of Malaga, 29071 Malaga, Spain

**Keywords:** additive manufacturing, fused filament fabrication, surface roughness, thermoplastic polymers, ANOVA, Taguchi

## Abstract

The aim of this work was to conduct a dimensional study, in terms of microgeometry, using parts from an additive manufacturing process with fused filament fabrication (FFF) technology. As in most cases of additive manufacturing processes, curved surfaces were obtained via approximation of planes with different inclinations. The focus of this experimental study was to analyze the surface roughness of curve geometry from surface-roughness measurements of the plane surfaces that generate it. Three relevant manufacturing parameters were considered: layer height, nozzle diameter and material. Taguchi’s experimental design based on the Latin square was applied to optimize the set of specimens used. For the manufactured samples, the surface-roughness parameters Ra (roughness average), Rq (root mean square roughness) and Rz (maximum height) were obtained in eight planes of different inclinations (0° to 90°). The results were analyzed using both a graphical model and an analysis of variance study (ANOVA), demonstrating the dependency relationships among the parameters considered and surface finish. The best surface roughness was reached at 85°, with a global average Ra value of 8.66 µm, increasing the average Ra value from 6.39 µm to 11.57 µm according to the layer height increase or decreasing it slightly, from 8.91 µm to 8.41 µm, in relation to the nozzle diameter increase. On the contrary, the worst surface roughness occurred at 20°, with a global average Ra value of 19.05 µm. Additionally, the theoretical profiles and those from the surface-roughness measurement were found to coincide greatly. Eventually, the eight regression curves from the ANOVA allowed prediction of outputs from future specimens tested under different conditions.

## 1. Introduction

By using a three-dimensional model with computer-aided design (CAD), additive manufacturing (AM) allows creation of objects via building them layer by layer. With a wide range of materials and technologies, additive manufacturing has proven to be one of the most efficient alternatives to traditional manufacturing. The set of technologies involved in AM aim to achieve the characteristics required for printed parts, both in design features and mechanical performance (Martín et al. [1]), taking advantage of the possibilities that these processes offer: cost and waste reduction, design freedom, flexibility, printing-time reduction, use of a variety of materials, mechanical-property and surface-roughness improvement, etc. In this last aspect, unlike with original models, parts manufactured with fused filament fabrication (FFF) technology present an inherent superficial texture due to its distinctive layer-by-layer deposition (Buj-Corral et al. [2], Delfs et al. [3]), which can be considered a surface-finish defect. Deposition, layer by layer, on a horizontal plane (XY) certainly produces different surface finishes depending on the surface inclination levels of the parts, which is clearly evident in curve geometry. Due to the difficulty of measuring the roughness of this type of surface, different angles are achieved through discretization in planes of different inclination values. 

The main objective of this work was to establish the influences of certain printing parameters on surface roughness according to the inclination-angle values of the surfaces that materialize curve geometry. 

In FFF technology, among the large number of parameters involved, layer height and nozzle diameter have determinant influences on the final geometry of a part. In addition, polymeric material was established as the third basic parameter of study in this work. Other parameters, such as deposition pattern, printing speed or printing temperature, were given secondary consideration. Consequently, to achieve the highest optimization level, experiments that allow for analysis of the influences of the principal printing parameters and satisfactorily predict the surface roughness that will result from the printing process need to be conducted. 

In this study, surface roughness was evaluated through means of measuring the relevant parameters, following the standard ISO 4287 [4]: roughness average (Ra), root mean square roughness (Rq) and maximum height (Rz). Even though this experiment resulted in higher values than were expected, understanding the influences of the printing variables considered is essential in order to minimize their negative effects on the surface quality of printed parts.

Galietto et al. [5] and Mendicky et al. [6] considered processing parameters such as deposition speed, layer height and infill density. Surface roughness was significantly influenced by these factors, according to the external geometry of the parts. As mentioned previously, layer height and nozzle diameter (hence deposited filament width) have different impacts on a curved external surface depending on its incline angle. In this case, different angles were achieved through discretization in planes of different inclination values. Alternative ways to minimize the roughness effect include, for example, surface chemical treatments (Taufik et al. [7]), which are difficult to control and create unacceptable problems in industrial components. This certainly affects the mechanical properties as well as the final dimensions and, for this reason, the tolerances of parts (Minetola et al. [8]). In the related literature, different aspects that influence roughness were considered. Vanaei H.R. et al. [9] and Bakrani et al. [10], for example, showed the effect of coalescence in deposited filaments caused by the temperature at the time of deposition and its cooling, with subsequent deformation of the filament section.

Many approaches have been considered to analyze these parameters’ influences. C. Lu [11] analyzed the effects of the machining-process parameters on surface-finish profiles. Wasserfall et al. [12] proposed an adaptive slicing process for layers height in order to reduce roughness. Urbanic et al. [13] suggested a theory study in which different kinds of deposited filament sections are contemplated. Di Angelo et al. [14] provided an alternative parameter to Ra using a theory analysis. However, no studies could be found in which the influence of the inclination angle of a surface studied was evaluated from both a theoretical and an experimental perspective and subjected to variation in the three main process parameters considered. Conclusions from Di Angelo [14] showed a highest roughness value at approximately a 20° inclination angle.

Although authors such as Kanta et al. [15] have worked with neuronal networks to study surface roughness, in this study, an analysis of variance (ANOVA) [16,17,18,19,20,21] is used. The application of this widely used method was first seen in the Taguchi design experiment (Mahmood et al. [22], Roy et al. [23]). In this method, to evaluate the influences of the parameters under study, the configuration is designed with three factors, and each factor is tested at three levels.

## 2. Materials and Methods

The thermoplastic polymers employed in this project for the AM process included PLA (polylactic acid), as in Koran et al. [24] and Kuznetsov et al. [25]; ABS (acrylonitrile butadiene styrene); and nylon 66 (PA-66) (Table 1). 

These materials were supplied by Ultimaker: Transparent PLA (code N/A), Black Nylon (code RAL 9011) and Grey ABS (code RAL 7011).

### 2.1. Design of Experiment

Test specimens for this study were printed using a 3D Ultimaker 2 Extended+ printer. This printer’s software configuration allows it to handle a large number of printing parameters, including layer height, deposition pattern, material, infill density and nozzle diameter. To achieve the robustness necessary for this study, it was essential to establish a suitable size for the samples. Consequently, taking into account the large number of parameters considered and the required sample size, an excessive number of specimens were initially needed. For this reason, optimization of the printing-parameter selection process was required. Following the premise that this study is focused on surface-finish evaluation and considering its geometric character, layer height and nozzle diameter were the main parameters used here. Additionally, due to sensitive differences in polymer printing conditions and behavior, material was established as the third factor to be considered.

Parameters such as deposition infill pattern and infill density were irrelevant, and therefore a grid infill pattern and an infill density of 20% were fixed for the whole set of specimens. 

The three main parameters and the others considered are included in (Table 2).

Print speed, top/bottom speed, print temperature and bed temperature values were obtained from the equipment specifications. 

**Figure 1:**
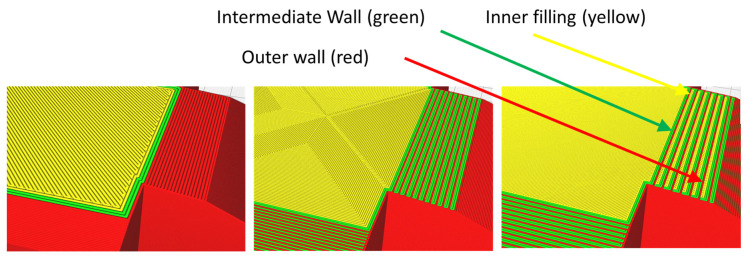
Examples of previous analysis. Red (outer wall), green (intermediate wall) and yellow (inner filling) layers.

Initially, a preliminary study was carried out through virtualization of a large number of possible combinations with which an approximation of the model could be determined, from a surface-finish point of view, depending on the different parameters applied. In all cases, the specimens consisted of several roof and floor layers, an intermediate layer and an internal structure with the selected deposition pattern and infill density. Afterward, it was verified that on the surfaces with reduced inclination (5° and 10°), the intermediate layer (green layer) between the outer wall (red layer) and the inner filling (yellow layer in Figure 1) were clearly perceptible, as these modified the configuration of the profiles subjected to measurement.

Based on this effect and in order to optimize the specimen-printing process, simulations were performed using all possible combinations of material, layer height and nozzle diameter, identifying in each case the affected planes, whose alteration varied depending on the different deposition patterns established with the intermediate layer. In this work, 9 3 × 3 matrices were defined, resulting in 81 study cases (Tables S1–S3).

From the results of the previous analysis, we found the following:

- With ABS (Table S1), all deposition patterns led to the formation of the yellow layer according to the inclined plane angle, this effect being clearly significant with the combination of the smallest nozzle diameter (d1 = 0.25 mm) and the largest layer height (h3 = 0.15 mm), since an inclination of up to 10° was observed in the measurement plane;
- Nylon (Table S2) presented this intermediate layer only at the 0° and 5° inclinations in different joints of diameter nozzle/layer height, using the same combinations as those applied for PLA and ABS;
- PLA (Table S3) presented exactly the same parameters as did ABS.

Data from the previous tables (Tables S1–S3) allowed us to conclude that all deposition patterns had the same low influence on the same planes (a maximum of 10°), except on nylon, whose greater expansion in the melting and deposition process was not affected by the 10° angle. Consequently, deposition pattern had a substantial effect on the 0° plane, which coincides with the material deposition plane, XY. For this reason, a linear pattern was established for all of the specimens.

Once this analysis was carried out, the values of the parameters in Table 2 were obtained, except for those from the deposition pattern, which was linear in all cases.

### 2.2. Design of the Part Geometry

In order to obtain a geometry that described a distinctly curved profile, the specimens included eight planes with different inclinations. Here, dimension was the main factor to consider. On one hand, printing time and material volume are variables directly influenced by defined specimen size. On the other hand, a minimum area is required to achieve a sufficient evaluation length. 

The eight study surfaces were materialized via eight inclined planes, 0°, 5°, 10°, 20°, 45°, 70°, 85° and 90°, with respect to the horizontal plane (XY). 

**Figure 2:**
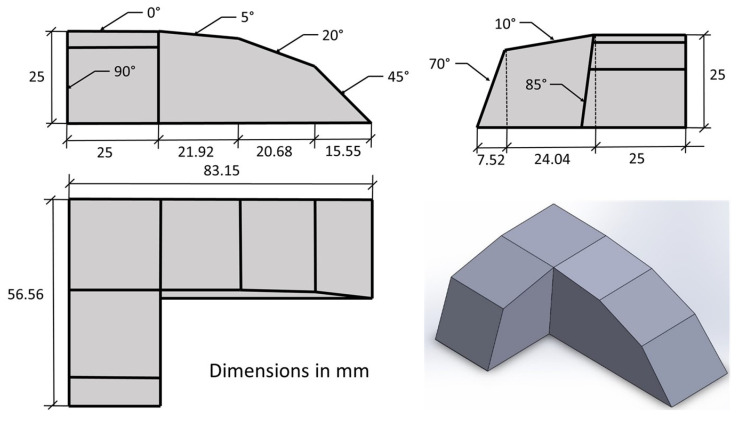
Specimen design with dimensions.

The specimens’ outer dimensions were 56.56 mm × 83.15 mm × 25 mm (height). Each plane had a measurement area of 550 mm^2^ (22 mm × 25 mm) (Figure 2).

### 2.3. Determination of Study Cases

From the combinations of the printing parameters previously defined, the initial number of study cases was determined to be 3^3^ = 27, with five measurements in eight different planes considered for each. Therefore, a total of 1080 registers were required. To optimize the sample size and meet the stated objectives, a Latin square design was applied [26]; this way, a minimum number of analyzed cases were needed to evaluate the influence of each parameter selected. Following this method, surface roughness was used as the response variable, expressed through the roughness parameters Ra, Rq and Rz. Factors included material (ABS (A), nylon (N) and PLA (P)), layer height (h), and nozzle diameter (d). This design only required 9 experimental units. The experimental layout was as follows (Table 3) (Figure S1):

### 2.4. Specimen Codification

Prior to the measuring process, each specimen was given a codification in order to determine its identity clearly and unambiguously. Each code generated was hxdxMx, where h, d and M identified the layer height, the nozzle diameter and the material, respectively. 

**Figure 3:**
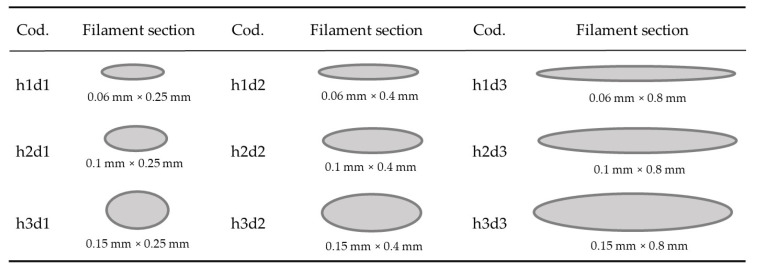
Theoretical elliptical sections of filaments.

The values for each parameter are provided in Table 2, and considering only the geometric parameters h and d, (Figure 3) shows a theoretical prediction of the geometries and dimensions of the filament sections deposited for each study case.

### 2.5. Specimen Measurement Process

The measuring equipment used in this work was the Mitutoyo Surftest SJ-210. This instrument includes the communication software SJ-Tools, which enables records that display assessed profiles and graphical data to be managed.

To guarantee accurate measurement on different planes, inclinations were fabricated using a gauge block set, a sine bar and a granite surface plate. 

Configuration of the evaluation parameters was established following the UNE-EN ISO 4287:1999 [4] and UNE-EN ISO 1302:2002 [27] standards. The measurement setting is defined as follows:

- Sampling length, lr = 0.8 mm;
- Evaluation length, ln = 4 mm;
- Total length of exploration = 4.8 mm.

Each of the 9 specimens was subjected to 5 measurements in the 8 considered planes of 0°, 5°, 10°, 20°, 45°, 70°, 85° and 90° (Figure 2).

## 3. Results and Discussion

These results were analyzed from two approaches. The first is the graphical approach, which allows us to explain surface-roughness behavior depending on the angle of a measured plane. The second approach involves the design of experiments (DOE) methodology with the analysis of variance model (ANOVA). The ANOVA allowed information about the functional relationship between printing parameters to be obtained and, additionally, provided predictive equations. 

As an example, the tables below (Tables S4–S6) show the arithmetic mean and standard deviation statistics of the roughness parameters Ra, Rq and Rz of the eight measured angles for three of the nine combinations derived from the Latin square. In these three cases, the layer height was fixed at 0.06 mm and nozzle diameters and materials were modified.

**Figure 4:**
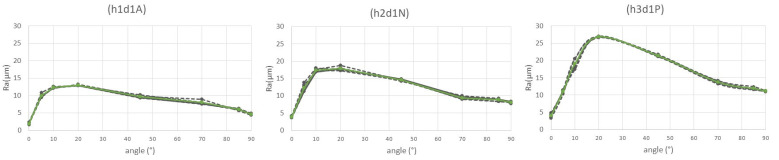
Ra average (green line) in h1d1ABS, h2d1 nylon and h3d1PLA.

Considering the other conditions, the nozzle diameter was established as 0.25 mm and layer heights and materials were set at the other values selected (Figure 4).

**Figure 5:**
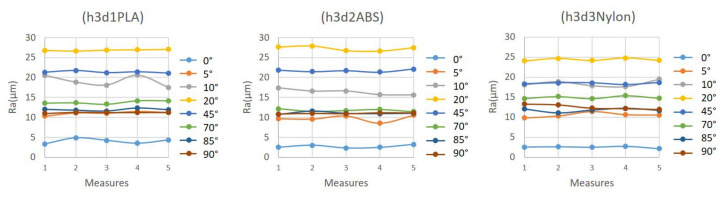
Ra values measured on eight angles of specimens h3d1PLA, h3d2ABS and h3d3 nylon.

The next figure (Figure 5) shows the low dispersion between the five measurements of the three samples. It can be observed that lines joining the values in the graphic are practically horizontal, except for in exceptional situations, even with minimum variations.

Standard deviations were also reduced, with the exception of occasional cases where models were not clearly defined and had values that reached a maximum of 1.45 µm (Figure S2).

**Figure 6:**
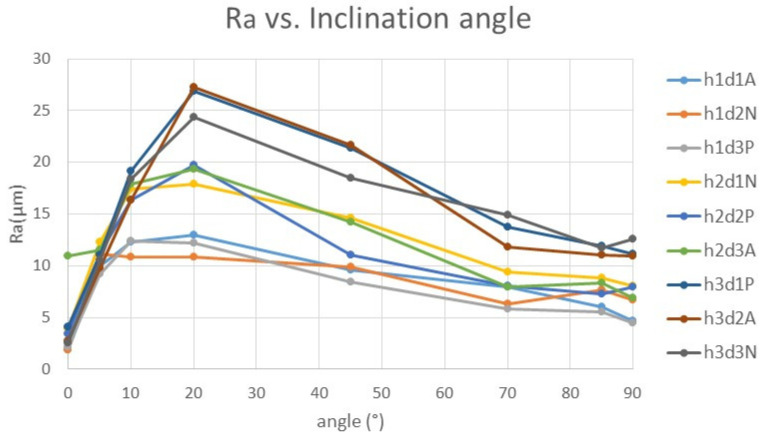
Average Ra values according to the angle of each surface measured.

**Figure 7:**
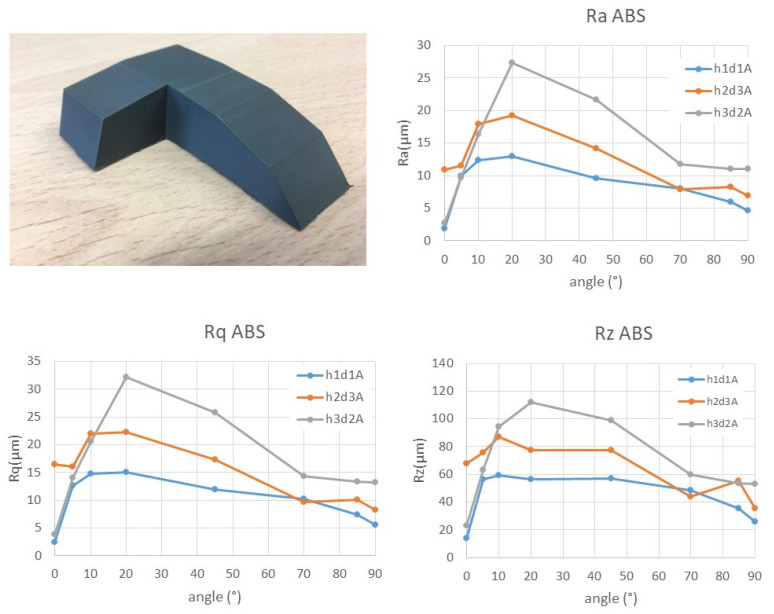
Average Ra, Rq and Rz values for the ABS material.

Initially, it was expected that due to the predictable roughness value in the horizontal plane (0°), it would increase up to the maximum value in the vertical plane, since this angle (90°) should have presented greater deviations when the roughness measurement was perpendicular to the deposition plane and to the elliptical section considered, always showing a greater depth in the spaces generated between layers. However, once the results were analyzed, in all cases studied, which were very different from each other, shape relationship values between nozzle diameter and layer height of 1.67 up to 13.33 were achieved, and a maximum roughness value of 20° was obtained, regardless of the combination of layer height, nozzle diameter and material (Figure 6, Figure 7, Figures S3–S5 and Table S7). 

**Figure 8:**
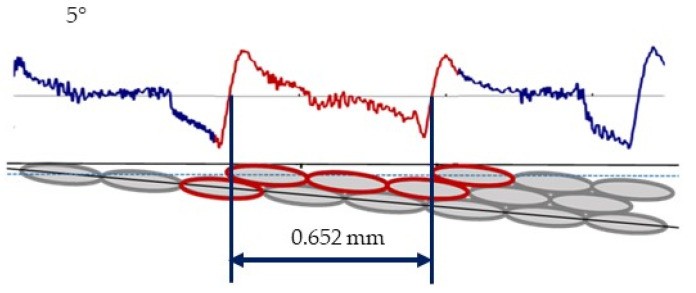
Real (**up**) and theoretical (**down**) profile of the 5° surface.

To explain this characteristic behavior, a comparison between the results from the rugosimeter and the theoretical values was drawn. Figure 8 shows the profile obtained from the rugosimeter measurement (blue color), with a section in the zone below the figure marked in red to represent the theoretical distribution for 5° (the downward inclination of the filament deposited).

**Figure 9:**
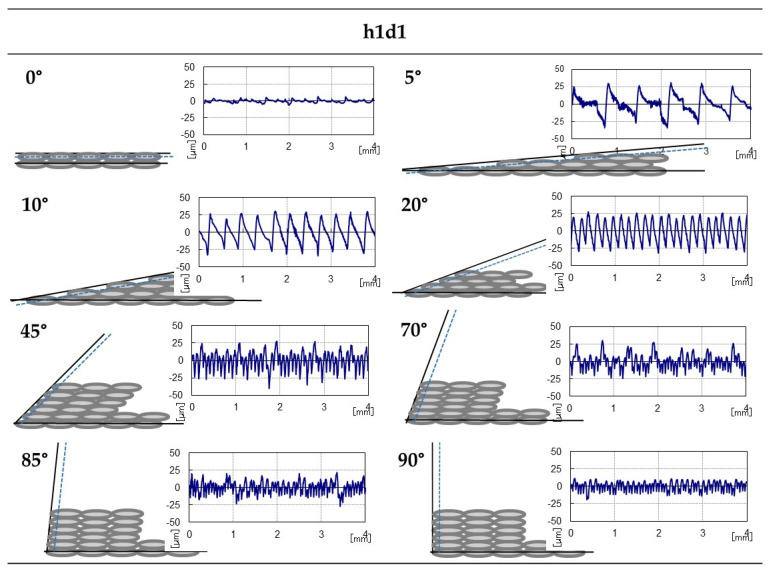
Theoretical (gray) and real (blue) profiles of the h1d1ABS specimen.

In extrapolation of this analysis to the set of measurements carried out, the same roughness pattern for each inclination was observed no matter the parameter combination selected. Taking a particular case into consideration, the next figure presents the h1d1ABS specimen’s profile from the rugosimeter and its theoretical profile (Figure 9). 

As is shown, the profiles from close measurement reflect the theoretical profile determined via the layer height and diameter nozzle parameters. Up to 20°, the “steps” of the profile decrease until disappearing completely. It is precisely this elimination of internal “steps” that provoked the greatest difference between the maximum and minimum values in a constant tendency without intermediate lower values. This situation generated the highest roughness in most of the studied cases (in any of the Ra, Rq and Rz parameters).

**Figure 10:**
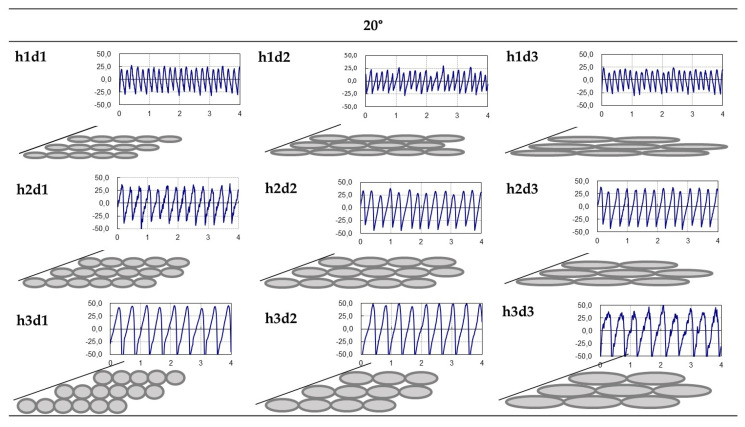
Theoretical (gray) and real (blue) profiles of the whole set of 20° specimens.

Considering that 20° is a critical angle and given that the highest values were obtained using this parameter, the whole set of nine specimens was analyzed. Figure 10 shows the real–theoretical profile pairs for all cases; here, it can be observed that the real profiles maintained the same pattern, with peaks and valleys without intermediate values.

Finally, considering the first approach established, we came to the conclusion that the 20° angle presented the maximum values of roughness for any combination of layer height, nozzle diameter and material. As surface roughness is a critical requirement, parts should be designed with appropriate inclinations. Di Angelo et al. [14] reached similar conclusions for other combinations, achieving the highest values at 27° and comparable evolutions in a set of inclinations of different surfaces.

Taking into account the second approach, a DOE with three factors (height layer, nozzle diameter and material) and three levels was conducted. Following the Taguchi method [23], this experiment was designed based on a Latin square of the order 3. Samples had a size of five registers (Table S8). Statgraphics software (Centurion, version 19) [28] allowed us to understand the influences of the input parameters (factors) on the response variable (Ra for the angles 0° (Var 1), 5° (Var 2), 10° (Var 3), 20° (Var 4), 45° (Var 5), 70° (Var 6), 85° (Var 7) and 90° (Var 8)) and, additionally, to assess the relationships between parameters through analysis of variance (ANOVA), regression equations and functions of multiresponse optimization. 

Tables S9–S11 summarize the analysis of variance established with the Statagraphics 19 software for the critical case of 20°.

The statistic R-square indicates that the model explains 96.758% of the variability at 20°.

Table S12 shows the arithmetic means for 20° at each factor level, as well as standard errors, offering a measurement of the variability of the samples. The last two columns present values that consider a confidence interval of 95% for each mean.

In Table S13, *p*-values under 0.05 (factor h) explain a possible correlation at a confidence interval of 95%. To simplify this model, since the *p*-value corresponding to the material factor was very high (0.5567) and therefore not statistically significant, with a confidence level of 95%, it could be eliminated.

Taking the coefficients from the previous table, the regression equation is

20° = 3.65821 + 156.995 × h + 1.23191 × d – 1.201 × Mat

Additionally, the coefficients of the other surfaces studied are shown in Table S14.

Table S15 shows the correlations between the coefficients of the adjusted model. There were no correlations with absolute values greater than 0.5 (the constant is not included). Consequently, there was no correlation between factors.

Developing the regression equations using values around those from the h1d1ABS combination, Figure S6 displays the evolution of the representative curve of the Ra values with d1 (0.25 mm) material (ABS) in relation to variable values of *h* in the 0.01–2 mm range.

This behavior study was extended to the nine regression equations from the surface map of each parameter combination for which the height-layer values corresponded to the nine selections of the Latin square applied. 

Figure S7 includes the nine graphics obtained. These were compared with those obtained directly from the means of the measurements in each study case.

In the figure above, a considerable similarity between the results from the regression equations and those from the testing performed on the specimens defined in the experiment (h1d1A, h1d2N, h1d3P, etc.) can be observed. As previously established, the greatest differences were found at the 0° angle, for which the R-square value barely reached 22.87%, for all nine specimens (Table S16).

On the other hand, the evolution of the regression equations showed a maximum slope at 20° (hd1A specimen with a nozzle diameter of 0.25 mm, ABS material and a layer height in the 0.01 mm to 0.2 mm range). This highlights that layer height exerts considerable influence on surface roughness generated (Figure S8).

In an analogous situation to the previous, considering a nozzle diameter within the range of 0.05 to 1 mm and with the same layer height, the slopes were substantially smaller (Figure S9), indicating the much-less-decisive influence of the “d” factor except for in the equation’s behavior around 0°, where this influence was greater than that observed for the “h” factor studied. However, due to the specific characteristics of this surface, whose deposition pattern is different from any other, this behavior was determined to be irrelevant in the big picture.

## 4. Conclusions

From this study, the following conclusions were drawn. 

With regard to surface-roughness level, we can firmly state that the influence of the inclination angles of surfaces is decisively important, although, in contrast with the initial hypothesis, where a gradual roughness increase for up to a 90° angle was expected, because of the elliptical section of the filament deposited, the results obtained for all of the specimens studied, with all combinations of parameters, show different behaviors. 

The inclination angles that presented better finishes, in decreasing order, were (Ra values in brackets) 85° (8.623 µm), 70° (9.209 µm), 5° (10.658 µm), 45° (14.721 µm), 10° (15.454 µm) and 20° (19.367 µm). The 0° and 90° angles were excluded due to their specific geometric features. According to these results, 20° was determined to be the most unfavorable inclination angle in terms of roughness. Thus, in part designs, this restriction should be considered using a combination of two planes, one with a greater inclination (85° approx.) and the other with a small angle (5° approx.), to avoid angles near this value.

Next, taking into account the influences of the three considered printing parameters (layer height, nozzle diameter and material) on surface-roughness level, the following conclusions were drawn.

Layer height was the main determinant of the roughness of the specimens studied, provoking an increase in Ra value in direct relation to any increase in the layer height used. These values ranged from Ra = 7.958 µm for the set of specimens with h1 = 0.06 mm to Ra = 11.433 µm for specimens with h2 = 0.1 mm and up to Ra = 14.265 µm for specimens with h3 = 0.15 mm. An increase of 150% in layer height induced an increase of 147% in Ra. A possible alternative way to reduce Ra values with an inclination angle of around 20° would be to work with a lower layer-height value, which would reduce the roughness of that zone.

The second parameter considered was nozzle diameter, which presented average Ra values that were very close to each other. Consequently, it can be said that this parameter does not have a decisive influence on surface finish, although it is possible that this reduced effect is actually due to the significant influence of layer height. This parameter did not even present a slight upward or downward tendency according to any increase in value of nozzle diameter, so a behavior pattern could not be established.

The average values of Ra regarding the nozzle diameters were Ra = 11.543 µm (d1 = 0.25 mm), Ra = 10.904 µm (d2 = 0.40 mm) and Ra = 11.209 µm (d3 = 0.8 mm). The variation in these results barely reached 5.5%. 

Finally, regarding the third printing parameter selected, material, as already shown through the variance analysis, does not have special relevance to surface finish. The average Ra value reached for nylon specimens was 11.215 µm; for ABS, the value was 11.414 µm, and PLA presented an average Ra of 11.028 µm, which supposes a percentage variation of 3.38%. This reduced variation was probably a result of the inaccuracy of the manufacturing process. Nevertheless, the good plastic-deformation properties of the nylon material led to an improvement in its surface roughness under the conditions of the present study.

## Figures and Tables

**Table 1:** Mechanical and physical property values of thermoplastic-filament materials.

Material	Diameter (mm)	Young’s Modulus (MPa) ASTM D3039	Tensile Stress at Yield (MPa) ASTM D3039	Tensile Stress at Break (MPa) ASTM D3039	Elongation at Break (%) ASTM D3039	Hardness (Shore D) ISO 7619-1	Density (g/cm3) (ISO 1183, Nylon, ABS) (ASTM D1505, PLA)	Melting Temperature (°C) ISO 11357	Printing Temperature (°C)
Nylon	2.85 ± 0.05	2331 ± 55	63.1 ± 1.1	40.4 ± 2.2	>120	81	1.14	188.4	245
PLA	2.85 ± 0.10	3250 ± 119	52.5 ± 0.9	45.5 ± 1.1	7.8 ± 1.2	84	1.24	151.8	200
ABS	2.85 ± 0.10	1962 ± 31	38.1 ± 0.3	33.9 ± 1.5	4.6 ± 0.3	76	1.10	amorphous	230

**Table 2:** FFF printing parameters considered in the experiment.

Parameter	Values	Unit
Deposition Pattern	Concentric, Lines, Zigzag	-
Nozzle Diameter	0.25, 0.4, 0.8	mm
Layer Height	0.06, 0.1, 0.15	mm
Material	Grey ABS, Black Nylon, Transparent PLA	-
Print Speed	30 (ABS, PLA), 40 (Nylon)	mm/s
Top/Bottom Speed	20	mm/s
Print Temperature	230 (ABS), 245 (Nylon), 200 (PLA)	°C
Bed Temperature	80 (ABS), 60 (Nylon), 60 (PLA)	°C

**Table 3:** The 3 × 3 Latin square design.

		d (mm)		
		0.25	0.40	0.60
**h (mm)**	**0.06**	M1 (ABS)	M2 (Nylon)	M3 (PLA)
	**0.10**	M2 (Nylon)	M3 (PLA)	M1 (ABS)
	**0.15**	M3 (PLA)	M1 (ABS)	M2 (Nylon)

## Data Availability

Not applicable.

## Supplementary Materials

The following supporting information can be downloaded at: https://www.mdpi.com/article/10.3390/polym15030585/s1, Table S1. Preliminary study: Gray ABS. Affected planes; Table S2. Preliminary study: Black Nylon. Affected plane; Table S3. Preliminary study: Transparent PLA. Affected planes; Table S4. Ra, Rq and Rz values with h1d1ABS study case; Table S5. Ra, Rq and Rz values with h1d2Nylon study case; Table S6. Ra, Rq and Rz values with h1d3PLA study case; Table S7. Ra average values; Table S8. DOE values for ANOVA; Table S9. Factors for ANOVA; Table S10. Analysis of variance; Table S11. Summary of the model ANOVA; Table S12. Means and confidence intervals for ANOVA; Table S13. Coefficients of the 20° regression equation from ANOVA; Table S14. Regression equation coefficients from ANOVA; Table S15. Correlation matrix from ANOVA; Table S16. R-square vs angles; Figure S1. Specimens’ configuration according the 3 × 3 Latin square design; Figure S2. Examples of Standard deviations of Ra according to angles in three specimens; Figure S3. Average Ra, Rq and Rz values for Nylon material specimens; Figure S4. Average Ra, Rq and Rz values for PLA material specimens; Figure S5. Ra average values for each factor; Figure S6. Curves evolution with different *h* values and only one *d1A* combination; Figure S7. Ra values of regression equations by ANOVA vs. testing values; Figure S8. *hd1A* specimen vs *Ra* angles; Figure S9. *h1dA* specimen vs *Ra* angles.
