# Supplementary material for: Analysis of the Effect of the Surface Inclination Angle on the Roughness of Polymeric Parts Obtained with Fused Filament Fabrication Technology
Source: Polymers (Basel). 2023 Jan 23;15(3):585. doi: 10.3390/polym15030585 (PMC9919160; doi:10.3390/polym15030585)
Supplement: Supplementary file 1 [file polymers-15-00585-s001.zip › polymers-2132040-supplementary.pdf]

polymers

Supplementary Materials

Supplementary Tables

Table S1. Preliminary study: Gray ABS. Affected planes.

	Gray ABS								
	Lines			Concentric			Zigzag		
	d1	d2	d3	d1	d2	d3	d1	d2	d3
**h1**	0º	0º	0º	0º	0º	0º	0º	0º	0º
**h2**	0º, 5º	0º, 5º	0º	0º, 5º	0º, 5º	0º	0º, 5º	0º, 5º	0º
**h3**	0º, 5º, 10º	0º, 5º	0º	0º, 5º, 10º	0º, 5º	0º	0º, 5º, 10º	0º, 5º	0º

Table S2. Preliminary study: Black Nylon. Affected plane.

	Black Nylon								
	Lines			Concentric			Zigzag		
	d1	d2	d3	d1	d2	d3	d1	d2	d3
**h1**	0º	0º	0º	0º	0º	0º	0º	0º	0º
**h2**	0º, 5º	0º, 5º	0º	0º, 5º	0º, 5º	0º	0º, 5º	0º, 5º	0º
**h3**	0º, 5º	0º, 5º	0º	0º, 5º	0º, 5º	0º	0º, 5º	0º, 5º	0º

Table S3. Preliminary study: Transparent PLA. Affected planes.

	Transparent PLA								
	Lines			Concentric			Zigzag		
	d1	d2	d3	d1	d2	d3	d1	d2	d3
**h1**	0º	0º	0º	0º	0º	0º	0º	0º	0º
**h2**	0º, 5º	0º, 5º	0º	0º, 5º	0º, 5º	0º	0º, 5º	0º, 5º	0º
**h3**	0º, 5º, 10º	0º, 5º	0º	0º, 5º, 10º	0º, 5º	0º	0º, 5º, 10º	0º, 5º	0º

Table S4. Ra, Rq and Rz values with h1d1ABS study case.

	(h1d1ABS)					
	Ra (µm)		Rq (µm)		Rz (µm)	
Plane	Arithmetic Mean	Standard Deviation	Arithmetic Mean	Standard Deviation	Arithmetic Mean	Standard Deviation
**0º**	1.90	0.27	2.43	0.33	13.90	1.89
**5º**	9.94	0.56	12.63	0.60	56.49	2.93
**10º**	12.34	0.17	14.79	0.25	59.52	1.88
**20º**	12.95	0.18	15.01	0.18	56.30	1.32
**45º**	9.64	0.34	12.00	0.46	56.86	2.19
**70º**	7.98	0.53	10.16	0.64	48.41	3.95
**85º**	6.00	0.26	7.34	0.35	35.25	3.31
**90º**	4.63	0.19	5.62	0.23	25.96	2.59

Table S5. Ra, Rq and Rz values with h1d2Nylon study case

	(h1d2Nylon)					
	Ra (µm)		Rq (µm)		Rz (µm)	
Plane	Arithmetic Mean	Standard Deviation	Arithmetic Mean	Standard Deviation	Arithmetic Mean	Standar Deviation

**0º**	1.83	0.07	2.27	0.08	11.32	0.55
**5º**	11.09	0.54	13.61	0.83	62.34	8.90
**10º**	10.89	0.50	12.65	0.55	48.05	1.19
**20º**	10.83	0.08	12.73	0.08	50.44	1.06
**45º**	9.88	0.18	12.09	0.27	54.33	1.32
**70º**	6.34	0.54	8.28	0.35	39.75	1.79
**85º**	7.62	0.82	9.55	0.94	46.65	5.37
**90º**	6.68	0.68	7.86	0.82	35.09	2.79

Table S6. Ra, Rq and Rz values with h1d3PLA study case.

	**(h1d3PLA)**					
	**Ra (µm)**		**Rq (µm)**		**Rz (µm)**	
**Plane**	**Arithmetic Mean**	**Standard Deviation**	**Arithmetic Mean**	**Standard Deviation**	**Arithmetic Mean**	**Standard Deviation**
**0º**	2.21	0.40	2.92	0.54	18.43	5.84
**5º**	9.25	0.16	11.96	0.16	53.66	1.67
**10º**	12.44	0.35	14.69	0.35	57.08	1.86
**20º**	12.19	0.18	14.05	0.20	52.11	1.42
**45º**	8.42	0.23	10.52	0.35	52.93	4.15
**70º**	5.87	0.40	7.57	0.44	37.81	2.68
**85º**	5.57	0.11	6.79	0.16	32.19	1.82
**90º**	4.51	0.14	5.49	0.22	25.46	2.41

Table S7. Ra average values.

**Angle**	**h1d1A**	**h1d2N**	**h1d3P**	**h2d1N**	**h2d2P**	**h2d3A**	**h3d1P**	**h3d2A**	**h3d3N**	**Average**
0º	1.895	1.832	2.207	3.915	3.388	10.899	4.066	2.698	2.698	**3.733**
5º	9.942	11.091	9.248	12.279	11.266	11.566	11.042	9.744	9.744	**10.658**
10º	12.337	10.892	12.438	17.435	16.314	17.861	19.099	16.353	16.353	**15.454**
20º	12.946	10.833	12.187	17.894	19.744	19.296	26.863	27.272	27.272	**19.367**
45º	9.635	9.877	8.420	14.574	11.025	14.219	21.341	21.698	21.698	**14.721**
70º	7.982	6.339	5.867	9.412	8.040	7.953	13.756	11.766	11.766	**9.209**
85º	6.004	7.623	5.572	8.813	7.270	8.296	11.921	11.056	11.056	**8.623**
90º	4.632	6.684	4.513	8.090	7.921	6.914	11.155	10.977	10.977	**7.985**
Average	**8.171**	**8.146**	**7.556**	**11.551**	**10.621**	**12.125**	**14.905**	**13.945**	**13.945**	**11.219**

Table S8. DOE values for ANOVA.

**h**	**d**	**Mat**	**Obs.**	**Var 1**	**Var 2**	**Var 3**	**Var 4**	**Var 5**	**Var 6**	**Var 7**	**Var 8**
1	1	A	1	1.720	9.453	12.368	12.993	9.393	7.761	6.298	4.815
1	1	A	2	1.837	10.040	12.126	13.241	9.807	8.883	5.628	4.417
1	1	A	3	2.293	9.665	12.225	12.822	10.150	8.030	6.108	4.439
1	1	A	4	1.603	10.876	12.576	12.822	9.344	7.656	5.851	4.730
1	1	A	5	2.023	9.676	12.390	12.852	9.482	7.584	6.135	4.761
1	2	N	1	1.949	10.342	10.458	10.785	9.879	6.560	8.098	7.256
1	2	N	2	1.797	10.933	10.307	10.744	9.716	5.464	8.400	7.039
1	2	N	3	1.796	11.309	11.464	10.823	9.718	6.570	7.575	6.284
1	2	N	4	1.847	11.827	11.228	10.958	10.142	6.218	6.269	5.676
1	2	N	5	1.771	11.045	11.004	10.855	9.932	6.886	7.776	7.169
1	3	P	1	2.647	9.186	12.496	11.924	8.285	6.330	5.420	4.760
1	3	P	2	2.467	9.067	12.595	12.326	8.722	5.743	5.619	4.446

1	3	P	3	1.892	9.415	11.831	12.091	8.301	5.873	5.652	4.402
1	3	P	4	1.696	9.166	12.597	12.364	8.604	5.283	5.491	4.478
1	3	P	5	2.336	9.407	12.673	12.232	8.190	6.108	5.681	4.482
2	1	N	1	4.199	11.994	17.685	18.754	14.441	9.260	8.384	8.137
2	1	N	2	4.208	13.836	17.996	17.583	14.744	9.000	8.389	8.251
2	1	N	3	3.714	13.070	17.306	17.308	14.504	9.053	8.960	8.013
2	1	N	4	3.601	11.269	17.267	18.082	14.274	10.020	9.305	7.696
2	1	N	5	3.853	11.229	16.924	17.747	14.911	9.729	9.028	8.354
2	2	P	1	3.525	9.619	16.902	19.502	11.019	8.392	7.560	7.753
2	2	P	2	3.166	13.162	16.855	19.262	11.064	8.198	7.294	8.169
2	2	P	3	3.131	10.129	15.978	19.856	11.294	8.020	7.002	7.666
2	2	P	4	3.843	12.191	15.863	20.074	10.828	7.741	7.402	7.889
2	2	P	5	3.275	11.233	15.974	20.026	10.921	7.851	7.095	8.132
2	3	A	1	10.690	11.719	17.820	19.353	14.503	8.271	8.069	6.981
2	3	A	2	12.040	10.425	17.956	19.673	14.010	8.131	8.591	6.893
2	3	A	3	9.884	11.878	17.888	19.749	13.716	8.046	8.417	6.814
2	3	A	4	10.980	12.033	18.237	18.856	14.244	7.514	8.494	6.918
2	3	A	5	10.903	11.775	17.406	18.851	14.625	7.805	7.912	6.965
3	1	P	1	3.345	10.323	20.524	26.802	21.308	13.573	12.008	10.957
3	1	P	2	4.889	11.125	18.818	26.649	21.719	13.645	11.760	11.241
3	1	P	3	4.246	11.052	18.095	26.891	21.188	13.303	11.559	11.182
3	1	P	4	3.515	11.493	20.562	26.934	21.400	14.140	12.378	11.186
3	1	P	5	4.339	11.220	17.500	27.039	21.093	14.122	11.903	11.209
3	2	A	1	2.504	9.689	17.379	27.639	21.831	12.178	10.727	10.800
3	2	A	2	2.990	9.584	16.560	27.885	21.479	11.514	11.590	10.953
3	2	A	3	2.335	10.377	16.582	26.751	21.717	11.730	11.001	10.875
3	2	A	4	2.469	8.534	15.660	26.618	21.347	11.970	10.932	11.145
3	2	A	5	3.196	10.537	15.588	27.470	22.117	11.440	11.031	11.112
3	3	N	1	2.534	9.790	18.108	24.070	18.317	14.671	12.016	13.289
3	3	N	2	2.612	10.291	18.881	24.664	18.568	15.152	11.012	13.117
3	3	N	3	2.503	11.445	17.886	24.177	18.562	14.623	11.724	12.251
3	3	N	4	2.710	10.644	17.648	24.773	18.146	15.377	12.195	12.143
3	3	N	5	2.161	10.549	19.476	24.174	18.636	14.709	11.666	11.932

Table S9. Factors for ANOVA.

Factor	Type	Levels	Values
h	Fixed	3	1; 2; 3
d	Fixed	3	1; 2; 3
Mat	Fixed	3	1; 2; 3

Table S10. Analysis of variance.

	DF	SS Adjust.	MS Adjust.	F-Value	p-Value
h	2	301.657	150.829	12200.88	0.003
d	2	0.824	0.412	33.35	0.029
Mat	2	8.233	4.116	332.98	0.000
Error	2	0.025	0.012		
Total	8	310.739			

Table S11. Summary of the model ANOVA.

S	R-square	R-square (adjusted)
0.110997	96.758%	96.528%

Table 12. Means and confidence intervals for ANOVA.

Level	Cases	Arithmetic Mean	Standard Error	Lower Limit	Upper Limit
Media Global	9	19.0454			
Factor h					
1	3	11.9887	0.0641927	11.7125	12.2649
2	3	18.9783	0.0641927	18.7021	19.2545
3	3	26.1693	0.0641927	25.8931	26.4455
Factor d					
1	3	19.2347	0.0641927	18.9585	19.5109
2	3	19.2833	0.0641927	19.0071	19.5595
3	3	18.6183	0.0641927	18.3421	18.8945
Factor Mat					
1	3	19.8383	0.0641927	19.5621	20.1145
2	3	17.7000	0.0641927	17.4238	17.9762
3	3	19.5980	0.0641927	19.3218	19.8742

Table S13. Coefficients of the 20° regression equation from ANOVA.

Parameter	Coefficient	Standard Error	T-Value	*p*-Value
Constante	3.65821	0.725231	5.04399	0.000
h	156.995	4.49415	034.9332	0.000
d	−1.23191	0.712783	−1.72831	0.0915
Mat	−1.201	2.02653	−0.59264	0.5567

Table S14. Regression equation coefficients from ANOVA.

Angle	Coef.	h	d	Mat
0º	2.712	9.404	4.080	−9.718
5º	10.872	2.693	−1.045	0.509
10º	8.123	65.207	0.800	2.167
20º	3.658	156.995	−1.232	−1.201
45º	4.186	125.096	−2.409	−7.943
70º	2.068	75.994	−0.723	−0.064
85º	3.211	57.875	−0.613	−0.987
90º	0.666	70.156	−0.230	1.778

Table S15. Correlation matrix from ANOVA.

	Constant	h	d	Mat
Constant	1.0000	−0.6403	−0.4750	−0.5588
h	−0.6403	1.0000	0.0000	0.0000
d	−0.4750	0.0000	1.0000	0.0000
Mat	−0.5588	0.0000	0.0000	1.0000

Table S16. R-square vs angles.

Angle	0º	5º	10º	20º	45º	70º	85º	90º
R-square (%)	22.8775	5.2927	69.3985	96.758	94.6567	84.565	88.0934	90.5954

Supplementary Figures

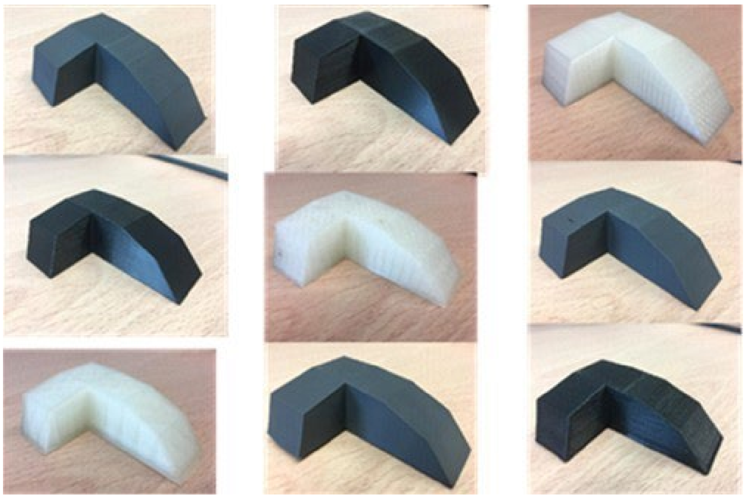

Figure S1. Specimens' configuration according the 3 × 3 Latin square design.

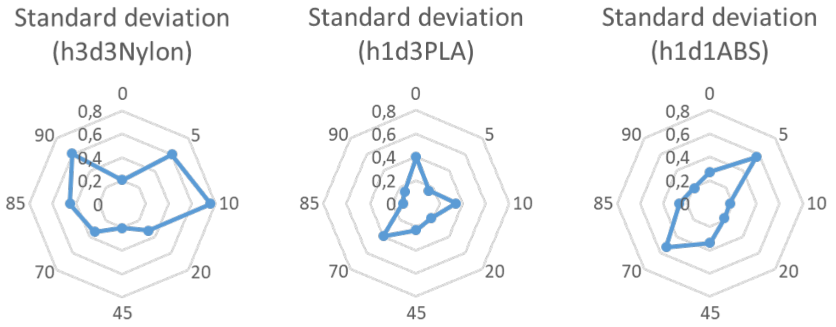

Figure S2. Examples of Standard deviations of Ra according to angles in three specimens.

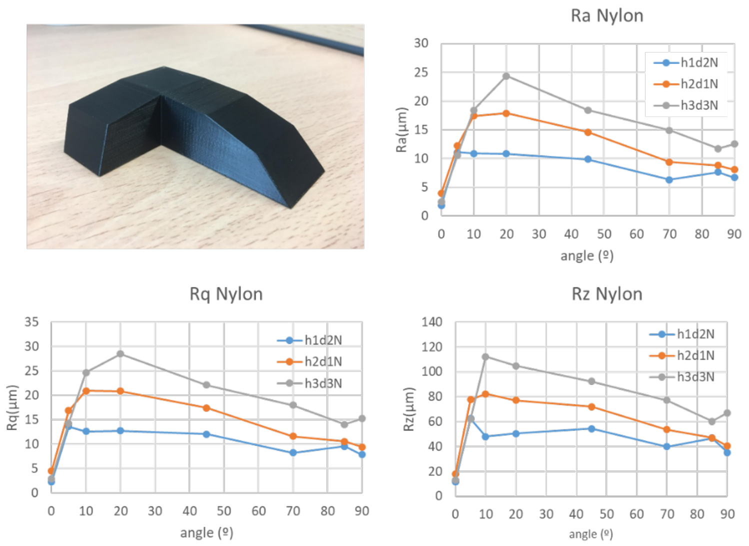

Figure S3. Average Ra, Rq and Rz values for Nylon material specimens.

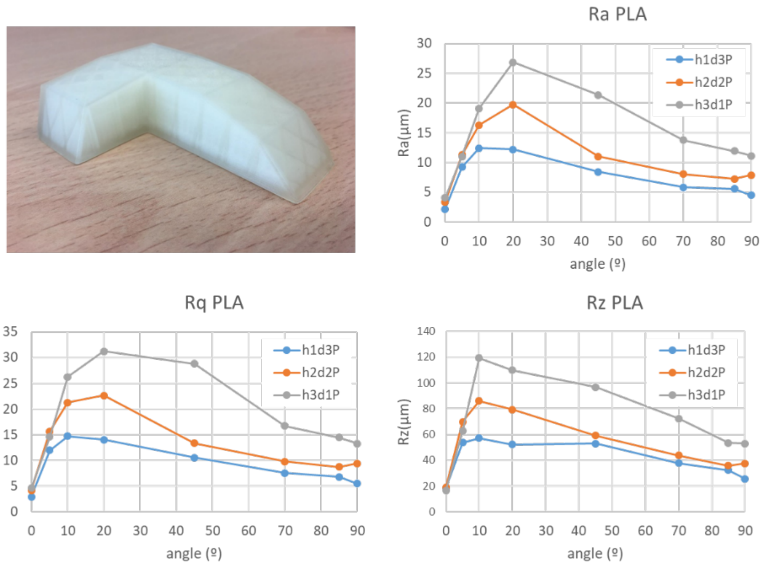

Figure S4. Average Ra, Rq and Rz values for PLA material specimens.

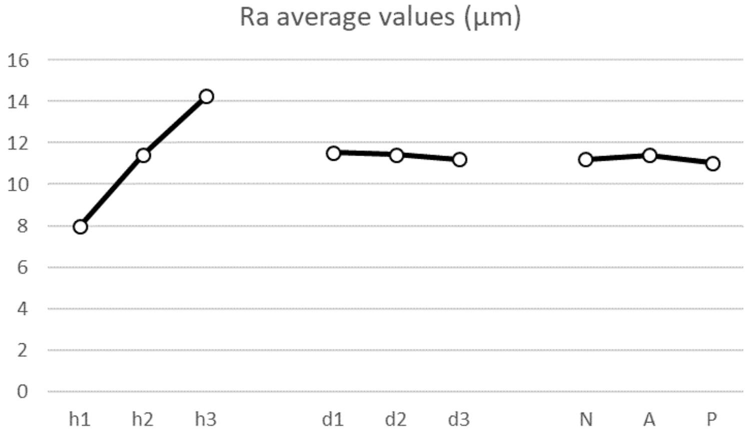

Figure S5. Ra average values for each factor.

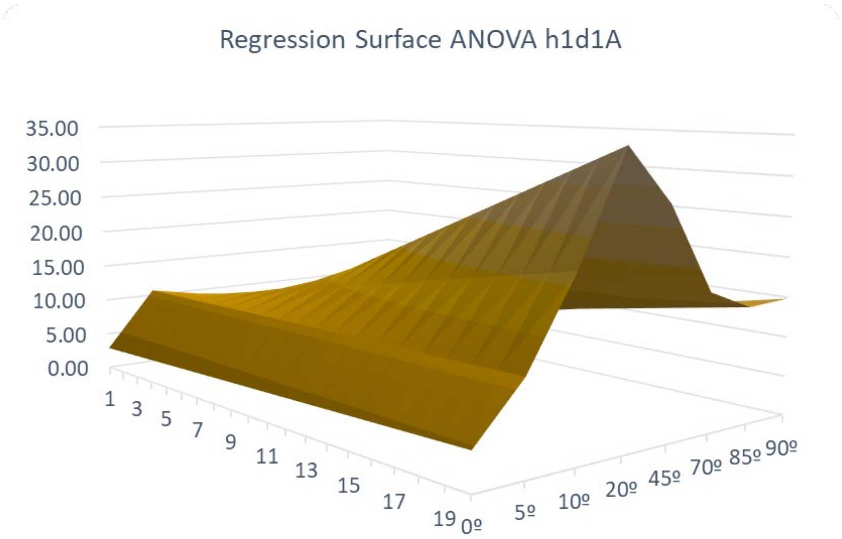

Figure S6. Curves evolution with different *h* values and only one *d1A* combination.

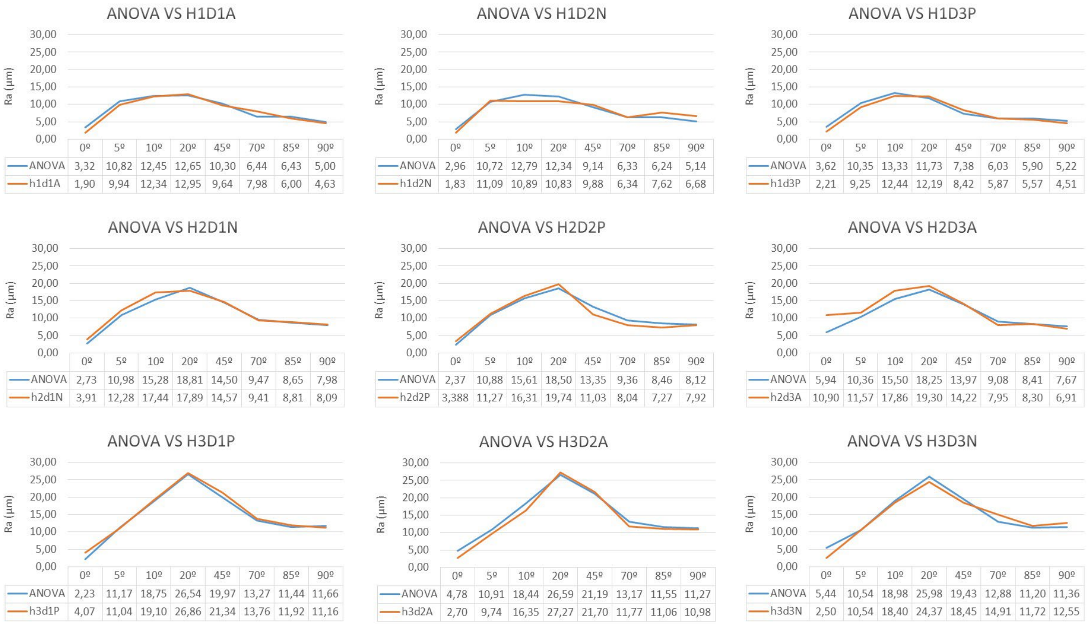

Figure S7. Ra values of regression equations by ANOVA vs. testing values.

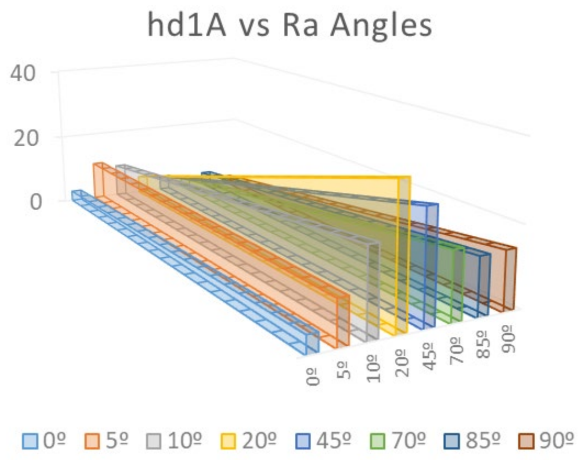

Figure S8. *hd1A* specimen vs *Ra* angles.

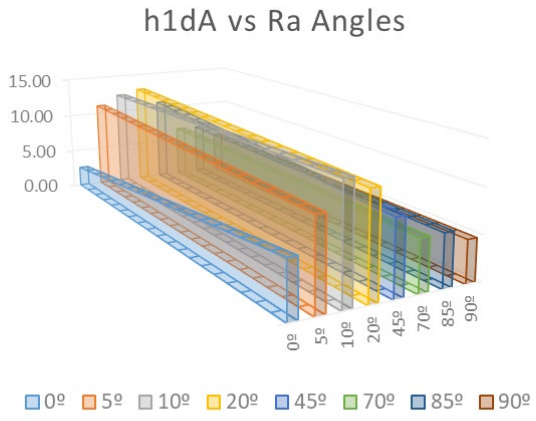

Figure S9. *h1dA* specimen vs *Ra* angles.
